# Self-Determination in People with Intellectual Disability: The Mediating Role of Opportunities

**DOI:** 10.3390/ijerph17176201

**Published:** 2020-08-26

**Authors:** Eva Vicente, Cristina Mumbardó-Adam, Verónica M. Guillén, Teresa Coma-Roselló, María-Ángeles Bravo-Álvarez, Sergio Sánchez

**Affiliations:** 1 Department of Psychology and Sociology, University of Zaragoza, C./Pedro Cerbuna, 12, 50009 Zaragoza, Spain; marian@unizar.es; 2 Psychology and Educational Sciences Studies, Open University of Catalonia, Rambla del Poblenou, 156, 08018 Barcelona, Spain; cmumbardoa@uoc.edu; 3 Department of Education, University of Cantabria, Av./de los Castros, 52, 39005 Santander, Spain; guillenvm@unican.es; 4 Department of Education, University of Zaragoza, C./Pedro Cerbuna, 12, 50009 Zaragoza, Spain; tcoma@unizar.es; 5 Department of Developmental and Educational Psychology, Autonomous University of Madrid, C./Francisco Tomas y Valiente, 3, 28049 Madrid, Spain; sergio.sanchezfuentes@uam.es

**Keywords:** intellectual disability, self-determination, opportunities, mediation analysis

## Abstract

The Convention on the Rights of Persons with Disabilities have proclaimed the basic right of people to make one’s own choices, have an effective participation and inclusion. Research in the field of disability have stressed self-determination as a key construct because of its impact on their quality of life and the achievement of desired educational and adulthood related outcomes. Self-determination development must be promoted through specific strategies and especially, by providing tailored opportunities to practice those skills. Providing these opportunities across environments could be especially relevant as a facilitator of self-determination development. This manuscript aims to ascertain if opportunities at home and in the community to engage in self-determined actions are mediating the relationship between people intellectual disability level and their self-determination. Results have confirmed direct effects of intellectual disability level on self-determination scores. Indirect effects also predicted self-determination and almost all its related components (self-initiation, self-direction, self-regulation, self-realization, and empowerment) through opportunities in the community and at home. Autonomy was predicted by the intellectual disability level through an indirect effect of opportunities at home, but not in the community. These results highlight the need for further research to better operationalize and promote contextually rooted opportunities for people with intellectual disability to become more self-determined.

## 1. Introduction

Current trends in self-determination research and promotion highlight it as a key construct in the lives of people with intellectual disability (ID). For both adolescents and adults, self-determination related skills are determinant to face curricular, transition to adulthood, and also job-related challenges and situations [1], but also to stand for their right to live a life of quality and to reach personal goals. In fact, that self-determination related skills, such as choice-making, problem solving or goal-setting and goal-attainment skills are useful abilities that serve multiple goals and have a positive impact in people with disabilities life has largely been proved [2]. These abilities are not only useful to navigate daily life situations and challenges, but perhaps more importantly, are necessary to fight for one’s rights such as living independently, being included and fully participate in one’s community or having access to information so as to take well-informed decisions, as described in the Convention on the Rights of Persons with Disabilities [3]. For this main reason and given the importance and scope of self-determination related components in the lives of people with disabilities, self-determination research has constantly endeavored to better understand its development so as to build responsive contexts for people to engage in self-determined actions.

Research under the Causal Agency Theory has propelled a deeper understanding of self-determination development by reconceptualizing it according to the newest knowledge stemming from positive psychology frameworks and from the strengths-based approach through which disability is currently understood [4]. Under this framework, self-determined action is embodied and operationalized by three essential characteristics: volitional action, agentic action, and action-control beliefs [5]. Volitional action refers to the extent to which a person makes intentional, conscious choices based on individual preferences and interests. Agentic action involves self-directing and managing actions in service of a freely chosen goal and implies identifying different ways to solve a problem, engaging in self-directed action, and managing, self-regulating and evaluating the actions taken. In being engaged in volitional and agentic actions, people develop adjusted action-control beliefs about their own performance and abilities [6]. Action-control beliefs include control-expectancy, that is believing one’s skills and resources will enable goal attainment, psychological empowerment, and self-knowledge of strengths and weakness to reach goals. When people act in a self-determined manner engaging in volitional and agentic actions mediated by action-control beliefs, they respond to environmental challenges (opportunities or threats); thus, propelling self-determination to develop [6]. Causal Agency Theory defines self-determination “as a dispositional characteristic manifested as acting as the causal agent in one’s life” [5] (p. 257). In this sense, self-determination must be understood as a tendency to act in a certain way, which is a frame of reference through which a person evaluates a situation and acts accordingly. Importantly though, this personal tendency might not be wrongly assimilated to a static trait, but it is contrarily shaped by contextual variables both across and within individuals, as this disposition interacts with situational characteristics of contexts that can either propel or thwart self-determined actions [6].

For this main reason, self-determination must not be understood in isolation of the context where self-determined actions occur, but must instead be comprehended across contexts that can have an impact, influence and be part of self-determination development. The opportunities provided in those contexts may act as catalyzers or barriers of self-determined actions [7]. As facilitating elements, opportunities provide context embedded situations to put into practice self-determination related skills, which implies evaluating and gauging the appropriateness of engaging in self-determined actions and acting in a self-determined way by navigating challenges as they occur. For instance, in educational contexts, it has been proved that, besides teaching self-determination related skills, instruments as the Self-Determined Model of Instruction [8] may guide the provision of opportunities to practice these skills along the school day and in a wide array of learning situations, maximizing thus self-determination related learning [9]. Too often though, people with disabilities are barely provided with enough opportunities in the community [10], at home or at school [11]. Professionals working with adolescents, but also with adults with intellectual disability, claim for further opportunities for them to engage in self-determined actions at a microsystem level, but perhaps more importantly, at a macrosystem level as well, to maximize its effect in learning to act in a self-determined manner [7]. In fact, teaching people self-determination related skills and providing them with opportunities to act in this way in the contexts where they live or develop (e.g., home, school job places, and community) are undoubtedly helpful strategies but still not enough sustainable ones. To this aim, social environments need to warrant mechanisms for people with intellectual disability to act as causal agents in their communities as well.

Given the importance of self-determination and its promotion, it is important to explore the impact of both personal characteristics and contextual variables on people self-determination. These personal and contextual variables are likely to serve as predicting, moderating, and mediating variables influencing the effect of self-determination and, as such, must be considered in the design and implementation of specific interventions. Several studies have identified personal characteristics that are associated with self-determination. Research has shown that students with ID are less self-determined than their peers without disabilities [12], or with learning and other disabilities [13], and that there is a positive correlation between intellectual functioning, as measured by IQ tests or other estimations, and self-determination [14,15,16]. However, research has also shown that other factors, particularly environmental and contextual factors, are stronger predictors of self-determination status than intellectual level [15,17,18], suggesting that all individuals can enhance self-determination when appropriate supports [19] and opportunities to engage in self-determined actions are provided. Wehmeyer and Bolding [20,21] demonstrated that the placement where a person with ID disability lived or worked (i.e., restrictive or inclusive setting) strongly predicted higher or lower self-determination. The provision of opportunities to engage in self-determined actions might though be influenced by contextual variables. For instance, in congregate or restrictive settings, opportunities to choose and act as the causal agent in one’s life are likely to be restricted [16]. These findings clearly underline that while intellectual functioning may be related with the level of support a person will need to become fully self-determined, the degree to which this person will finally engage in self-determined actions basically depends on the opportunities provided in his or her environment and the supports available to succeed in this environment [22].

There is a clear need to investigate the specific role of opportunities in these contexts where people live or develop as catalyzers of self-determination, given the different roles and influences that these opportunities might have on self-determination development. For instance, opportunities at home may have a strong impact in adolescents with intellectual disability volitional actions, whereas opportunities at school influenced action control beliefs [23]. Moreover, the impact of contextual opportunities on self-determination dimensions differed according to the presence of disability. Higher support needs, and specifically the intellectual disability level, has traditionally been associated with self-determination, with lower levels of self-determination being reported for people with severe and profound intellectual disability [24]. Seeing people as more or less capable of acting in a self-determined manner can clearly determine the frequency and quality of opportunities given in their contexts; thus, aligning with an understanding of the context as interactive amongst multilevel (involving micro, meso, and macro level) and multifactor variables such as culture or family embedded conceptions and dynamics [25]. Setting the environment for the person with intellectual disability to act in a self-determined manner might not be enough if the levels and factors influencing opportunities are not identified and manipulated to enhance self-determination. Following Schalock, Luckasson, and Shogren [25], in order to identify and create appropriate opportunities, several models such as quality of life, human rights, human functioning and multidimensional context model should be combined together. However, to the best of our knowledge, there is still scarce research on this topic, and given its complexity, an in-depth approach of the role of environmental opportunities in self-determination development urges. In line with recent studies that have recognized the relevance of testing moderator and/or mediator variables that may be influencing people with disabilities quality of life [26], the aim of this study is to ascertain if opportunities to engage in self-determined actions are mediating the relationship between the person ID level and their level of other-reported self-determination, that is, if opportunities to act in a self-determined manner actually explain the relationship amongst intellectual disability and self-determination levels. Our results will have thus the potential to better inform the design and implementation of interventions to promote self-determination [22].

## 2. Materials and Methods

### 2.1. Participants

As self-determination of people with intellectual disability was not reported by themselves but by professionals working with them, throughout this section we will refer to the former as participants and to the latter as informants. The recruitment criteria were (1) people with intellectual disability; and (2) being aged between 11 and 40 years. No exclusion criteria were established. The final sample of participants included 541 evaluated participants with intellectual disability, the majority being men (*n* = 334; 61.7%), and with an average age of 26.28 years (SD = 8.28). The distribution according to gender and age was non-homogeneous ($\chi_{\left(30\right)}^{2}$ = 47.23, *p* < 0.05). Information about age, gender, and the level of intellectual disability is provided in Table 1. Furthermore, informants were required to provide associated conditions and possible specific etiologies. Behavioral problems were reported in 25.9% (*n* = 140) and communication problems in 18.1% (*n* = 98); motor disability 16.3% (*n* = 88); epilepsy 14% (*n* = 76); autism spectrum disorder up to 8.7% (*n* = 47); cerebral palsy 6.9% (*n* = 32); visual impairment 3.7% (*n* = 20); and hearing impairment 2.4% (*n* = 13). It should also be noted that in 12.2% of the sample (*n* = 66), intellectual disability was associated with Down syndrome. Participants information was gathered through an initial questionnaire (see below).

The sample recruitment was incidental, with the voluntary participation of multiple organizations. Professionals of these institutions and organizations collected the data, only when potential participants (or their legal representatives) provided informed consent to participate.

The assessment was carried out by 181 informants from 33 Spanish agencies that provide support to people with intellectual and developmental disabilities. In this case, the number of women (75.1%) was higher in comparison to the number of male informants (24.9%). All were professionals, with diverse profiles, including teachers (21%), professional caregivers (20.4%), psychologists (9.9%), occupational therapists (3.9%), directors of centers or services (2.8%), speech therapists (2.8%), social workers (2.8%), and educators (2.8%). Informants had known participants for at least four months (with a mean of five years and four months). Almost all of them (74.8%) had daily contact with the participant or at least several times per week.

### 2.2. Measures

#### 2.2.1. Sociodemographic Data

An initial questionnaire was used to collect sociodemographic data from both respondents and participants, including variables such as gender and age. Informants also had to classify the appropriate participants severity level of ID giving an estimate of their intellectual functioning (i.e., mild, moderate, severe, and profound) based on the current International Statistical Classification of Diseases [27] at the moment when the study was held. Informants were asked to use available reports in their organizations with information about Intellectual Quotient (IQ) or other clinical judgments to classify participants.

#### 2.2.2. Self-Determination Opportunities

For the purpose of this study, a brief scale was elaborated to measure opportunities for self-determination, with 12 items divided into two subscales that measure their opportunities at (1) home (six items) and (2) in the community (six items) to perform self-determined actions. These subscales gathered data on the other-reported opportunities to engage in self-determined actions. Scores are rated on a Likert scale from 1 (Never) to 4 (Always). Items details and psychometric properties of the instruments are in the first results part.

#### 2.2.3. Personal Self-Determination

The AUTODDIS Scale is addressed to assess self-determination of adolescents and adults from 11 to 40 years old with ID. The scale is composed of six subscales, which can be assembled into three domains of self-determination according to the most recent theoretical model [5]. The first domain, volitional characteristics (autonomous and volitional actions), is made up of two subscales: autonomy (7 items) and self-initiation (6 items). The second domain, agentic characteristics (self-managed actions), includes the subscales of self-direction (12 items) and self-regulation (3 items). Finally, the action-control beliefs domain is structured around two subscales: self-realization (6 items), and empowerment (12 items). All items must be answered in a four-point Likert scale based on level of agreement (i.e., strongly disagree, disagree, agree, and strongly agree) by an external respondent who knows the person with ID well (for at least 4 months).

This scale has been developed following a solid elaboration process based on (1) a Delphi method [28]; (2) a pilot study [29]; and (3) a rigorous analysis of the reliability and validity evidences [30,31]. The final version of the scale shows Cronbach’s alpha values close to or greater than 0.95 as well as evidences of concurrent validity [31]. Results also confirmed the internal structure of this instrument and its equivalence and measurement invariance in adolescents and adults [30].

### 2.3. Procedures

The collection of information has been carried out with the collaboration of 33 organizations working with people with intellectual disabilities spread over 11 of the 17 autonomous communities in Spain. Since the objective was for the largest number of organizations to participate, in addition to sending an email invitation directly, the study was disseminated on the website of the Institute for Community Integration (INICO) of the University of Salamanca. Subsequently, the research team contacted the reference person at each participant organization and continued support was established for the research process.

The Ethics Committee of the Community of Aragon (CEICA) approved this study, indicating that it complied with the principles for research development set out in the Declaration of Helsinki. The research team kept all the informed consents that were collected through participating organizations. Identification codes were used, replacing the name and surname, to guarantee confidentiality and anonymity.

It is important to highlight the organizations valued contribution to the process, as their knowledge of participants eligibility to take part of the study was crucial to identify potential participants. Given that it is a third-party evaluation, organizations had also a relevant role in assigning a professional to act as informant. This informant had to know the person well (for at least 4 months with frequent contact) and be familiar with the self-determination construct.

Data collection consisted of three blocks: firstly sociodemographic data was collected; then informants should provide information about opportunities at home and in the community of the participants to engage in self-determined actions, and; finally they had to complete the AUTODDIS Scale. Participants’ organizations could decide upon online or paper format data collection. Most of them were conducted online (88%) and only 12% on paper.

### 2.4. Data Processing and Analyses

**Figure 1:**
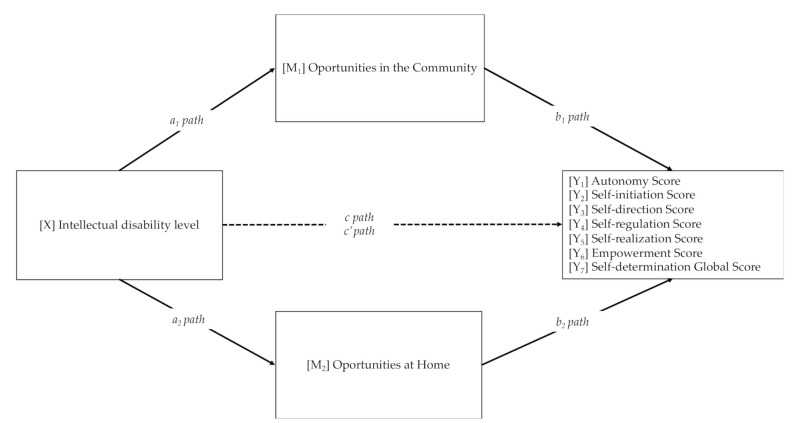
Conceptual diagram of the mediation analysis. Note: a_1_ path = effect of X on M_1_; b_1_ paths = effect of M_1_ on Y_i_; a_2_ path = effect of X on M_2_; b_2_ paths = effect of M_2_ on Y_i_; c paths = total effect of X on Y_i_; and c′ paths = direct effect of X on Y_i_ controlling for M. Seven separate paths were conducted (Y_1–4_) with the predictor (X).

Previous to main analysis, psychometric properties of the Self-Determination Opportunities instrument were reported. Then, in a first stage, descriptive statistics, internal consistency scores and correlations among study variables were examined. Correlations were determined to be weak (r < 0.30), moderate (r = 0.30–0.60), or strong (r > 0.60) based on established criteria [32]. In the second stage, preliminary associations between sociodemographic variables (i.e., gender and age range) and opportunities and level of self-determination were explored to identify confounding variables for statistical control before the main analyses. Mann–Whitney *U* and Kruskal–Wallis *H* tests (nonparametric tests for continuous variables) were carried out at this stage to examine differences in study variables by gender and age range. For the main analyses, several simple mediation models (model 4) of the PROCESS Macro version 3.3 by Andrew F. Hayes for SPSS [33] were performed to examine the direct relationship between ID level and self-determination and their components (measured with AUTODDIS Scale), as well as the indirect effect of opportunities at home and community on these relationships. These models reflect a causal sequence in which X_1_ affects Y_n_ indirectly through mediator variables M_n_ (see in Figure 1). The data analytic strategy utilized [34,35] allows for estimation and significance testing of the total indirect (mediation) effects through bootstrapping. Bootstrapping generates an empirical representation of the sampling distribution of the indirect effect from which a confidence interval can be generated [34]. Conservative confidence intervals (99%) were specified to adjust for Type I error rate inflation [36]; the confidence intervals (CIs) for the indirect effects were estimated with bootstrapped analyses (10,000 resamples) as recommended [34,35]. In other words, this conditional process analysis (CPA) produces CIs, based on bootstrapped sampling distribution, and it can be assumed that the indirect effects are significant, and that mediation occurs if zero falls outside the 99% confidence interval [37].

## 3. Results

### 3.1. Psychometric Properties of Self-Determination Opportunities Instrument

To guarantee the reliability of the Self-Determination Opportunities instrument, internal consistency was assessed by calculating Cronbach’s alpha and item-subscale correlation matrix was performed to identify low-discrimination items. The scale showed strong internal consistency (Cronbach’s alpha = 0.955). In turn, data yielded good values for Opportunities in the Community (0.958) and at Home (0.933) subscales. All items showed significant item-subscale correlations higher than 0.60 (ranging from 0.677 to 0.841).

In terms of validity, an exploratory factor analysis based on covariances matrix was conducted, using the principal axis factoring as a retention factor criteria and oblique rotation (oblimin). The aim was to check the dimensionality of the scale and the items factor loading, considering the following criteria: (a) explained variance based on eigenvalues; and (b) the rule 0.40, 0.30, and 0.20 [38]. Results supported a two-factor structure explaining 81.32% of the instrument variance, and all items following the rule 0.40, 0.30, and 0.20 and loaded in the proposed factor (Table 2).

### 3.2. Descriptive Statistics, Internal Consistency and Correlations

Descriptive statistics, correlations, and internal reliability scores are presented in Table 3. Internal reliability scores among study variables were deemed excellent (range = 0.842–0.982). Pearson and Spearman correlation analyses revealed moderate and strong associations (all significant, *p* < 0.01) among all variables. As expected, ID level was negatively correlated with all variables, showing a moderate inverse relation with the opportunities at home and community; and strong inverse associations with self-determination subscales and scale.

### 3.3. Preliminary Analysis

In terms of contrast statistics, nonparametric tests (i.e., Mann–Whitney *U* and Kruskal–Wallis *H* tests) were used to identify confounding variables for statistical control before conducting the main analyses (Table 4). These analyses revealed self-determination subscales and opportunities scores were not associated with gender, except for the autonomy score. Males and females showed similar levels of self-initiation (*p* = 0.817), self-direction (*p* = 0.562), self-regulation (*p* = 0.456), self-realization (*p* = 0.440) and empowerment (*p* = 0.900). However, females showed more autonomy than males *(p* = 0.005). Regarding self-determination opportunities, no significant differences were found by gender (at home, *p* = 0.359; and in the community, *p* = 0.524).

Secondly, the analyses revealed that opportunities at home and community, as well as the scores in two of the self-determination subscales (i.e., self-regulation and self-realization) were not associated with age ranges. In contrast, participants between 22 and 30 years old reported more autonomy (*p* = 0.009) and self-direction (*p* = 0.014) levels than the other two age ranges; and more self-initiation (*p* = 0.006) and empowerment (*p* = 0.042) than younger ones.

Therefore, to control for any possible effects, age range and gender were entered as covariates in the subsequent mediation (Table 4).

### 3.4. Mediation Analysis

As depicted in Figure 1, seven multiple mediation analyses were conducted with the ID level as a dependent variable (X) and opportunities at home (M_1)_ and in the community (M_2_) as mediators. The independent variables were the six subscales of the AUTODDIS Scale (autonomy, self-initiation, self-direction, self-regulation, self-realization, and empowerment) and the global self-determination score (Y_1_–Y_7_). Indirect effects results are presented in Table 5.

In terms of the autonomy score, the results of this first mediation analysis showed that the full model with opportunities at home and community was significant (R^2^ = 0.396, F(4, 443) = 56.95, *p* < 0.001). The standardized parameter estimates of this model included a statistically significant direct effect of ID level on the autonomy score (i.e., c_1_ = −2.631, SE = 0.276, CI (−3.467, −1.917)). Testing indirect effect, results indicated that ID level was associated with the autonomy score, which indirectly occurred through the effect of the opportunities at home (i.e., a_2_*b_2_ = −0.657, SE = 0.157; CI (−1.102, −0.308)), but no through the effect of the opportunities in the community (i.e., a_1_*b_1_ = −0.190, SE = 0.144; CI (−0.575, 0.191)).

For the rest of the subscales scores, the full models with opportunities at home and in the community were significant (R^2^ ranged from 0.318 to 0.512, all *p* < 0.001). The standardized parameter estimates included a statistically significant direct effects of ID level on the self-initiation score (i.e., c_2_ = −1.571, SE = 0.192, CI (−2.067, −1.076)); self-direction score (i.e., c_3_ = −3.696, SE = 0.394, CI (−4.715, −2.678)); self-regulation score (i.e., c_4_ = −0.725, SE = 0.111, CI (−1.015, −0.440)); self-realization score (i.e., c_5_ = −1.310, SE = 0.187, CI (−1.793, −0.827)); and empowerment score (i.e., c_6_ = −4.084, SE = 0.360, CI (−5.015, −3.154)). The test of the indirect effect indicated that ID level were associated with these self-determination subscales, which indirectly through the effect of both opportunities at home and in the community (Table 5).

Finally, with regards to Self-determination Global Score, the full model with opportunities at home and in the community as mediators accounted for significant variance (R^2^ = 0.541, F(5, 434) = 102.39, *p* < 0.001). Although direct effects of ID level on the self-determination score was also significant (i.e., c_7_ = −13.022, SE = 1.239, CI (−17.228, −10.916)), and, in terms of indirect effects, ID level predicted self-determination indirectly through opportunities at the community (i.e., a_2_*b_2_ = −3.368, SE = 0.701, CI (−5.237, −1.685)) and home (i.e., a_1_*b_1_ = −2.977, SE = 0.713, CI (−4.978, −1.347)).

## 4. Discussion

The current study contributed to the literature by examining whether the role of opportunities at home and in the community explained, in part, the relationship between ID and self-determination level and its components in people with ID. Consistent with the theorical framework, the results of the present study showed that when professionals’ reports were used, opportunities at home and in the community mediated the association between intellectual functioning and self-determination level, as well as between intellectual functioning and five self-determination components (i.e., self-initiation; self-direction; self-regulation; self-realization, and empowerment). In contrast, opportunities at home, but not in the community, mediated the association between ID level and autonomy. A higher level of ID was associated with lower opportunities at home (the *a_n_ path*), which in turn was associated with lower level of autonomy, self-initiation, self-direction, self-regulation, self-realization, empowerment, and the global self-determination score (the *b_n_ path*). Notably, the observed indirect effects (the a_n_* b_n_ path) were evident for all criterion variables, after adjusting for the influence of covariates (such as, age and gender). The same results have been found with opportunities in the community, except for autonomy subdomain.

These findings align with other studies that found a positive correlation between intellectual functioning and self-determination, but also affirmed that this relationship might be influenced and explained by other variables [14,16]. Research has shown that other factors, particularly contextual variables (i.e., instructional factors, support needs, or choice opportunities) act as stronger predictors of self-determination than the intellectual level. [15,16,17,18] made a discriminant function analysis of predictors of self-determination scores, showing that only perception of choice opportunity (from among four variables, including IQ) predicted membership in the group of participants with high self-determination scores. Likewise, previous studies [39,40,41,42,43] have also described opportunities for self-determination as significant predictors of self-determined actions. Interestingly, the autonomy domain has been shown to be influenced by opportunities at home but not in other environments, in line with recent studies where data was collected directly amongst participants with ID [23]; thus, stressing this shared perspective. Engaging in volitional actions, that is initiating autonomous actions (with the required supports), is the necessary first step for then regulating and directing agentic actions by navigating challenges and problems as they occur, while nurturing a personal sense of empowerment and self-knowledge about one’s skills and resources to attain goals (action-control beliefs). It makes then sense that closer environments of the person, such as home, might be promoting autonomous actions in first place, though further research is required to design opportunities, in the community, to act in an autonomous way as well. All in all, these considerations must be understood with caution and without assimilating the provision of opportunities to leaving the person at his or her expense, but rather to providing tailored supports for the person to act autonomously and in an agentic manner, watching over his or her safety.

Consistently with the aim of this study, the analysis of self-determination of people with ID and related personal and environmental variables should be considered from a more sophisticated perspective, rather than simply focusing on relationships. No previous studies have considered incorporating these factors into a mediational analysis. Identifying mediator variables allow for a more in-depth understanding of the process by which two variables are related [26], and thus guide the decision making processes towards interventions and programs improvement, but also to evaluate the process of change achieved through an intervention, among others [44,45]. That opportunities to engage in self-determined actions both at home and in the community stand as key components in self-determination promotion seems undeniable, in the light of the presented results. Further, more sustainable and tailored opportunities to act in a self-determined manner not only at a microsystem level, but also in the community is exactly what professionals working both with adolescents but also with adults with ID in Spain are claiming for [23]. In other words, self-determination promotion should start by enhancing opportunities (mediating variables) so as to cause changes in the expected outcome, which is in the level of self-determination. However, further work is needed to operationalize how these contextual opportunities have to be built, considering the interaction between the different levels and factors [25] in order to propel self-determination development.

Further, and perhaps more importantly, more severe levels of ID were associated with less opportunities both at home and in the community to act in a self-determined manner which, in turn, ended in lower levels of global self-determination and self-determination components. As previously stated, people with more limitations in intellectual functioning (and other competences such as adaptive behavior), and commonly with higher support needs, tend to be offered with less opportunities to make their choices and act in a self-determined manner [24]. However, conversely, people with higher support needs will probably need additional opportunities to practice self-determined related abilities in a wide array of environments to reach higher self-determination levels. People with higher support needs have the right to be provided with as much opportunities as needed to develop self-determination and its related components, and the environments where they live and develop embody the best scenarios for contextual opportunities onset. Further efforts must be then devoted to providing support to services providers, stakeholders, professionals and families for them to create contextualized and relevant opportunities for people with ID at home and in the community.

Despite its contribution to the existing body of knowledge and its implications on self-determination promotion, the present study is not exempt from some limitations. First, the study design was cross-sectional and correlational, and causality therefore cannot be discerned from the data. More randomized controlled trials are needed to test opportunities efficacy in improving self-determination of people with ID. In addition, longitudinal studies are required to determine the underpinnings of this relationship and its directionality and strength (i.e., ID level, opportunities and self-determination). Second, the present study measured ID level as an estimation reported by professionals, and although this represents a common practice in our context where ID assessments might not be homogeneously reported, results have to be interpreted taking it into account. We were not able to collect data on students’ levels of intelligence (IQ). Likewise, opportunities at home and community were measured as specific variables, using no standardized questionnaires. Although, data indicates that it possesses adequate reliability and validity; the understanding of contextual opportunities as the interaction amongst multiple levels and factors might require other measurement alternatives. The operationalization and evaluation of opportunities to engage in self-determined actions stand as a necessary line of future research in the field, as undoubtedly, these opportunities will need to vary and be tailored to the person developmental stage (early adolescence, transition to adulthood period, etc.). Third, a narrow set of variables were incorporated for analysis in terms of their influence on self-determination. We focused only on ID level and opportunities at home and in the community but, other personal and contextual variables may also play a mediational role on the development and expression of self-determination. More complex models should be tested to clarify how multiple factors may influence people with ID to become self-determined. Finally, the convenience sample was recruited from organizations that agreed to participate in this project, so the results might be interpreted within the context where the sample was drawn and taking into account it is not necessarily representative of the population of people with ID in Spain.

## 5. Conclusions

The mediating role of contextual opportunities (at home and in the community) was observed in the relationship between ID level and self-determination as well as most of their specific domains (i.e., self-initiation; self-direction; self-regulation; self-realization, and empowerment). Interestingly, the autonomy domain has been shown to be influenced by opportunities at home but not in the community. This study contributes to increase the understanding of self-determination of people with ID and related personal and environmental variables from a more sophisticated perspective, rather than simply focusing on relationships. Hopefully, these findings will have implications in fostering the incorporation of contextual opportunities into self-determination promotion and intervention programs.

## Figures and Tables

**Table 1:** Characteristics of participants (% (N)).

Variable		Participants (*n* = 541)			Total
Age Range		11−21 Years % (N)	22−30 Years % (N)	31−40 Years % (N)	
Gender	Male	66.7 (116)	57.8 (104)	61.3 (114)	61.7 (334)
	Female	33.3 (58)	42.2 (76)	38.7 (72)	38.1 (206)
	Missing Data	-	-	-	0.2 (1)
ID level	Mild ID	31.6 (51)	51.1 (92)	36.0 (67)	38.8 (210)
	Moderate ID	46.0 (75)	32.8 (59)	43.5 (81)	39.7 (215)
	Severe ID	17.2 (28)	12.2 (22)	14.0 (26)	14.1 (76)
	Profound ID	5.5 (9)	3.9 (7)	6.5 (12)	5.2 (28)
	Missing Data	-	-	-	2.2 (12)

**Table 2:** Exploratory factor analysis.

Exploratory Factor Analysis Results Item by Item		Factor 1	Factor 2
Eigenvalue		7.255	1.316
% of Variance		67.64	12.27
Cumulative % of Variance		67.064	79.92
ROTATED LOADING MATRIX		Factor 1	Factor 2
Item 1	He/She has opportunities in the community to express his/her own interests and wishes.	-	0.621
Item 2	He/She has opportunities in the community to set his/her own goals.	-	0.573
Item 3	He/She has opportunities in the community to learn about making plans to achieve his/her own goals.	-	0.748
Item 4	People in the community encourage him/her to carry out own plans.	-	0.884
Item 5	People in the community tell him/her if he/she is achieving own goals.	-	0.883
Item 6	People in the community give advice and encourage him/her to change plans if they are not working.	-	0.818
Item 7	He/She has opportunities at home to express his/her own interests and wishes.	0.762	-
Item 8	He/She has opportunities at home to set his/her own goals.	0.787	-
item 9	He/She has opportunities at home to learn about making plans to achieve his/her own goals.	0.897	-
Item 10	People at home encourage him/her to carry out own plans.	0.882	-
Item 11	People at home tell him/her if he/she is achieving own goals.	0.894	-
Item 12	People at home give advice and encourage him/her to change plans if they are not working.	0.943	-

**Table 3:** Descriptive statistics, internal reliability and Pearson and Spearman’s correlations.

Variables		AUT	SIN	SDI	SRE	REA	EMP	SDGS	OH	OC	ID
**SIN**	P	0.812	-	-	-	-	-	-	-	-	-
	S	0.764	-	-	-	-	-	-	-	-	-
**SDI**	P	0.767	0.792	-	-	-	-	-	-	-	-
	S	0.726	0.745	-	-	-	-	-	-	-	-
**SRE**	P	0.653	0.643	0.770	-	-	-	-	-	-	-
	S	0.618	0.590	0.725	-	-	-	-	-	-	-
**REA**	P	0.721	0.757	0.785	0.681	-	-	-	-	-	-
	S	0.639	0.672	0.741	0.628	-	-	-	-	-	-
**EMP**	P	0.757	0.808	0.862	0.728	0.799	-	-	-	-	-
	S	0.694	0.740	0.824	0.685	0.748	-	-	-	-	-
**SDGS**	P	0.877	0.895	0.943	0.800	0.871	0.944	-	-	-	-
	S	0.845	0.850	0.926	0.768	0.814	0.924	-	-	-	-
**OH**	P	0.475	0.588	0.488	0.400	0.444	0.488	0.545	-	-	-
	S	0.428	0.530	0.457	0.369	0.373	0.432	0.497	-	-	-
**OC**	P	0.424	0.535	0.544	0.463	0.454	0.532	0.562	0.692	-	-
	S	0.408	0.519	0.537	0.453	0.436	0.535	0.565	0.700	-	-
**ID**	P	−0.595	−0.580	−0.597	−0.485	−0.544	−0.660	−0.656	−0.314	−0.390	-
	S	−0.527	−0.503	−0.544	−0.424	−0.413	−0.556	−0.566	−0.269	−0.353	-
i		7	6	12	3	6	12	46	6	6	1
*n*		541	541	541	541	541	540	540	494	449	530
*Missing*		0	0	0	0	0	1	1	47	92	11
M		18.34	16.40	25.86	6.74	15.81	29.77	113.00	18.79	15.66	1.85
Median		19	17	26	7	16	31	117	20	16	2
SD		5.56	4.29	8.34	2.06	3.69	8.45	29.37	4.70	5.31	0.86
Min		7	6	12	3	6	12	46	6	6	1
Max		28	24	48	12	24	48	184	24	24	4
Sk		−0.458	−0.488	−0.054	−0.118	−0.622	−0.540	−0.500	−0.742	−0.006	0.791
Ku		−0.460	0.045	−0.432	−0.417	0.747	−0.168	−0.029	−0.342	−0.979	−0.037
α		0.916	0.903	0.961	0.841	0.909	0.949	0.982	0.933	0.958	-

Note: AUT = autonomy score; SIN = self-initiation score; SDI = self-direction score; SRE = self-regulation score; REA = self-realization score; EMP = empowerment score; SDGS = Self-determination AUTODDIS Global Score; HO = Opportunities at home Score; CO = Opportunities in the community Score; ID = Intellectual Disability Level; P = Pearson correlations; S = Spearman Correlation; i = number of items; n = sample; M = mean; SD; Standard Deviation; Min = minimum; Max = maximum; sk = symmetry; ku = kurtosis; α = Cronbach alpha.

**Table 4:** Contrast statistics.

	Age Ranges					Gender			
Variables	11–21	22–30	31–40	H	*p*	Female	Male	U	*p*
	M(SD)	M(SD)	M(SD)			M(SD)	M(SD)		
**AUT**	18.03(0.44)	19.59(0.43)	18.73(0.43)	9.416	0.009	18.92(0.28)	18.24(0.54)	29622.5	0.005
**SIN**	15.88(0.34)	17.31(0.33)	17.17(0.30)	10.321	0.006	16.86(0.21)	16.52(0.45)	34161	0.817
**SDI**	25.91(0.67)	27.79(0.66)	26.57(0.64)	8.519	0.014	26.79(0.42)	26.69(0.87)	33546	0.562
**SRE**	7.07(0.18)	6.97(0.16)	6.73(0.15)	2.099	0.350	6.90(0.10)	7.02(0.22)	33267.5	0.456
**REA**	15.88(0.32)	16.17(0.26)	16.27(0.26)	0.837	0.658	16.11(0.18)	16.09(0.38)	33215	0.440
**EMP**	29.53(0.68)	31.53(0.64)	31.14(0.58)	6.336	0.042	30.72(0.41)	30.86(0.85)	34180	0.900
**OH**	18.43(0.40)	19.01(0.40)	18.65(0.37)	1.595	0.450	18.82(0.24)	18.18(0.57)	27416.	0.359
**OC**	15.56(0.44)	16.01(0.45)	15.42(0.42)	0.777	0.670	15.68(0.27)	15.62(0.62)	22869.5	0.524

Note: AUT = autonomy score; SIN = self-initiation score; SDI = self-direction score; SRE = self-regulation score; REA = self-realization score; EMP = empowerment score; SDGS = Self-determination AUTODDIS Global Score; OH = Opportunities at home Score; OC = Opportunities in the community Score; M = mean; SD; Standard Deviation; H = Kruskal–Wallis H; U = Mann–Whitney U.

**Table 5:** Indirect effects.

M	Y	Mediators	Path	b	SE	LLCI	ULCI
M1	Y_1_	OC—Opportunities in the Community	(a_1_ * b_1_) ID-OC-AUT	*−0.190*	*0.144*	*−0.575*	*0.191*
		OH—Opportunities at Home	(a_2_ * b_2_) ID-OH-AUT	−0.657	0.157	−1.110	−0.308
M2	Y_2_	OC—Opportunities in the Community	(a_1_ * b_1_) ID-OC-SIN	−0.336	0.097	−0.593	−0.087
		OH—Opportunities at Home	(a_2_ * b_2_) ID-OH-SIN	−0.628	0.128	−0.972	−0.327
M3	Y_3_	OC—Opportunities in the Community	(a_1_ * b_1_) ID-OC-SDI	−1.224	0.249	−1.914	−0.617
		OH—Opportunities at Home	(a_2_ * b_2_) ID-OH-SDI	−0.606	0.185	−1.157	−0.189
M4	Y_4_	OC—Opportunities in the Community	(a_1_ * b_1_) ID-OC-SRE	−0.223	0.066	−0.429	−0.087
		OH—Opportunities at Home	(a_2_ * b_2_) ID-OH-SRE	−0.131	0.050	−0.280	−0.017
M5	Y_5_	OC—Opportunities in the Community	(a_1_ * b_1_) ID-OC-REA	−0.363	0.092	−0.613	−0.125
		OH—Opportunities at Home	(a_2_ * b_2_) ID-OH-REA	−0.293	0.097	−0.573	−0.076
M6	Y_6_	OC—Opportunities in the Community	(a_1_ * b_1_) ID-OC-EMP	−1.002	0.199	−1.563	−0.533
		OH—Opportunities at Home	(a_2_ * b_2_) ID-OH-EMP	−0.663	0.191	−1.201	−0.238
M7	Y_7_	OC—Opportunities in the Community	(a_1_ * b_1_) ID-OC-SD	−3.368	0.701	−5.237	−1.685
		OH—Opportunities at Home	(a_2_ * b_2_) ID-OH-SD	−2.977	0.713	−4.978	−1.347

Note 1: N for analyses is 541 cases. The standard error and 99% CI for the indirect effects (a * b) are obtained through bootstrapping with 10,000 re-samples. Note 2: b = Standardized Coefficients; SE = Standard Error; LLCI = lower 99% level confidence interval; ULCL = upper 99% level confidence interval; M_i_ = Mediation Models; (a_i_ * b_i_) = indirect effect; Y_1_ = AUT = autonomy score; Y_2_ = SIN = self-initiation score; Y_3_ = SDI = self-direction score; Y_4_ = SRE = self-regulation score; Y_5_ = REA = self-realization score; Y_6_ = EMP = empowerment score; Y_7_ = SD = Self-determination AUTODDIS Global Score; ID = Intellectual Disability Level. Note 3: Values in italics correspond to non-significant results
